# A narrative review of heterogeneity in SARS-CoV-2 infection outcomes and vaccine efficacy: strategizing pandemic preparedness in Africa

**DOI:** 10.3389/fpubh.2026.1761547

**Published:** 2026-02-03

**Authors:** Trisha Kerai, Mark Woolhouse, Norman Z. Nyazema, Francisca Mutapi

**Affiliations:** 1 Institute of Immunology and Infection Research, Ashworth Laboratories, King’s Buildings, University of Edinburgh, Edinburgh, United Kingdom; 2 Tackling Infections to Benefit Africa (TIBA) Partnership, TIBA Global Health Research Unit, Ashworth Laboratories, King’s Buildings, University of Edinburgh, Edinburgh, United Kingdom; 3 Usher Institute, University of Edinburgh, Edinburgh, United Kingdom; 4 Department of Biochemistry and Clinical Pharmacology, National University of Science and Technology, Bulawayo, Zimbabwe; 5 Department of Pharmacy, University of Limpopo, Sovenga, South Africa

**Keywords:** Africa, COVID-19, immune heterogeneity, infection susceptibility, pandemic preparedness, SARS-CoV-2, vaccine effectiveness, vaccine efficacy

## Abstract

Disease epidemiology during the COVID-19 pandemic differed greatly across the globe. In contrast to early pandemic predictions, Africa recorded the fewest SARS-CoV-2 related hospitalizations and deaths. Hypotheses proposed to explain this paradox include underreporting, age demographics, climate, national mitigation strategies, lifestyle factors, pre-existing cross-reactive protection, and host genetic determinants. This traditional, narrative review evaluates these hypotheses investigated in the published literature, and highlights knowledge gaps which limit our understanding and obscure validation of potential explanations. It also discusses how responses to vaccines, the primary intervention sought to control infectious disease outbreaks, may vary both within the African population and across other continents. Potential explanations in the literature include pre-existing immunity, poor nutrition, immune modulating co-infections, comorbidities, microbiome composition, genetic polymorphisms, and demographic factors. Previous studies have shown that pre-existing (infection-derived) immunity or cross-reactive immune responses can augment vaccine-elicited positive responses and can protect against reinfection in a way similar to immunization. Conversely, there are also studies showing that prior immunity interferes with the efficacy of new vaccines through mechanisms like original antigenic sin and immune imprinting. Thus, there is need for more immunology studies to understand the relative contribution of pre-existing cross-reactive immune responses to the epidemiology of new pathogens. These studies are particularly essential to understand the differences between pandemic preparedness and population vulnerability, as well as to inform vaccine development and vaccine effectiveness monitoring studies. SARS-CoV-2 serves as an important case study to understand heterogeneity between and within populations in immune responses to both the pathogen and to vaccination. This understanding is crucial in informing vaccine research and development aimed at supporting the 100-day mission for when the next pandemic threat emerges.

## 1 Introduction

**Figure 1:**
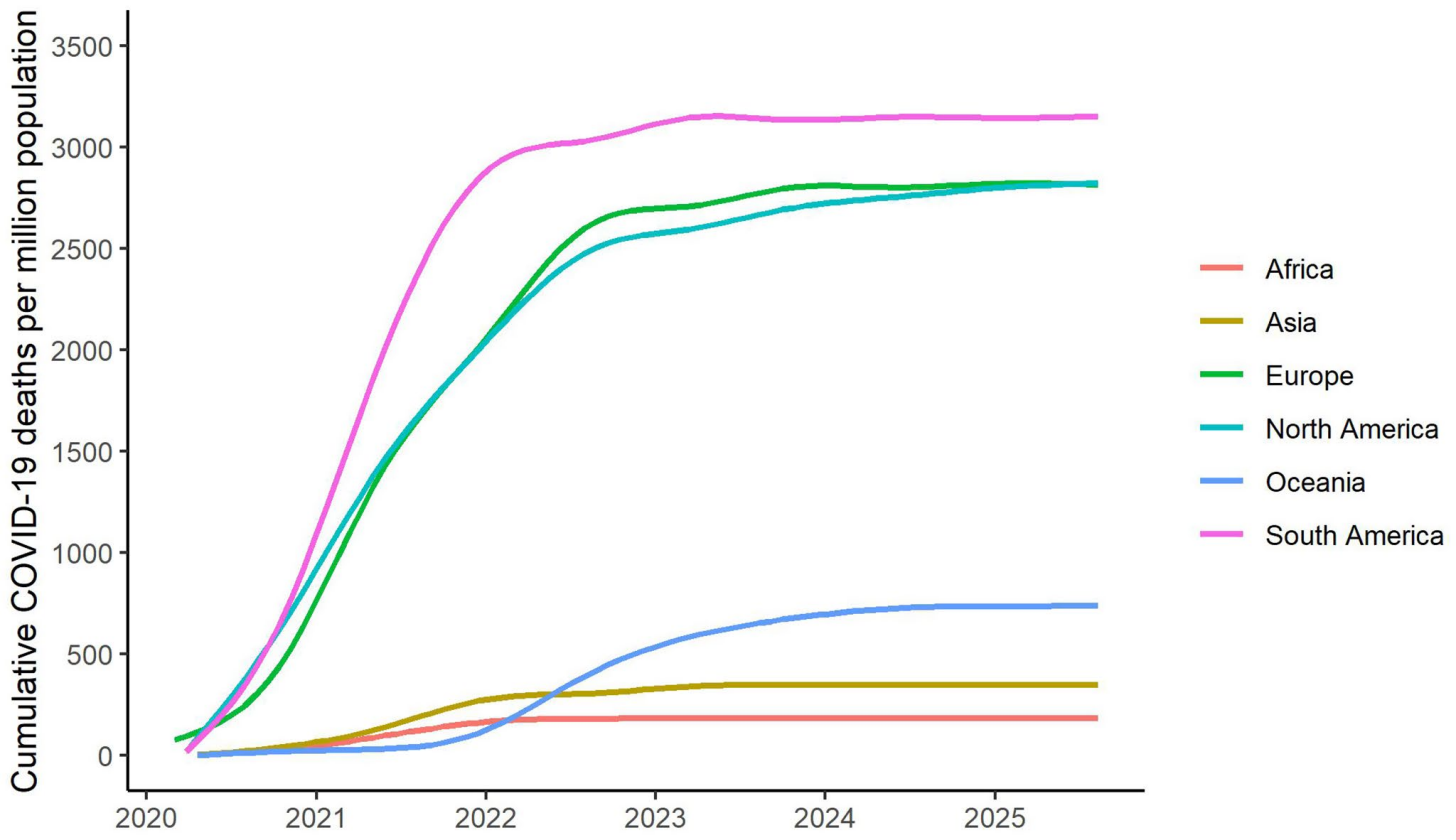
Cumulative confirmed COVID-19 deaths per million people by continent as of 10th August 2025. Source: our world in data.

Severe acute respiratory syndrome coronavirus 2 (SARS-CoV-2), a novel coronavirus first identified in December 2019 in Wuhan, China, was responsible for the COVID-19 pandemic (1). It has claimed the lives of over seven million individuals globally (2), and left many more debilitated (3). However, the burden of disease varied notably across regions, as highlighted in Figure 1, which illustrates the total number of deaths attributed to COVID-19 relative to population, and by continent (4). Contrary to some assumptions by scientists, which were primarily influenced by access to and quality of health systems among other factors, relatively fewer severe cases resulting in hospitalization and deaths were reported from Africa compared to other continents (5).

It is also important to note that while Africa stood out globally, responses within the continent were heterogenous. South Africa and most North African countries experienced more severe COVID-19 cases similar to those reported outside Africa (6, 7). The second wave also unfolded distinctly across the continent, as the Alpha variant drove a second surge in cases in North and West Africa, whilst the Beta variant dominated during the second wave in Southern Africa. However, around East and Central Africa, both variants circulated simultaneously (8).

Although the global disparity in SARS-CoV-2 infection outcomes has been widely acknowledged, there remains a paucity of data hindering our understanding of why this may have been the case. Particularly, lack of data on African populations’ immune responses to natural infection, and how infection history and resulting pre-existing heterologous immunity could have influenced immune defenses to SARS-CoV-2 in this population.

Multiple studies have analyzed SARS-CoV-2-specific cellular and humoral responses following vaccination and natural infection, but only a minority of participants included in these studies had an African background (9–11). A recent study has highlighted significant differences between a Senegalese and European cohort in both adaptive and innate immune responses to severe COVID-19 outcomes. Despite the Senegalese cohort having greater lymphopenia and more circulating neutrophils than the European cohort, the Senegalese cohort recruited fewer immune cell types overall (12). Another study conducted in Ghana also reported lower levels of cytokines associated with severe SARS-CoV-2 infection and COVID-19 pathology in SARS-CoV-2 positive individuals, compared to reports published from studies conducted in China and Belgium (13).

Additionally, as natural infection outcomes varied globally, responses to COVID-19 vaccines may have differed too. This has indeed previously been observed for multiple vaccines including attenuated malaria vaccines, the bacillus Calmette-Guerin (BCG) vaccine, and yellow fever vaccines (14). Table 1 summarizes key published review articles investigating factors affecting COVID-19 vaccine effectiveness.

**Table 1:** Factors affecting COVID-19 vaccine effectiveness.

Study (first author, year)	Study title	Study type	Summary of study findings	DOI (for source verification)
Bobrovitz N., 2023 (121)	Protective effectiveness of previous SARS-CoV-2 infection and hybrid immunity against the omicron variant and severe disease	Systematic review and meta-regression	Prior infection provides protection against reinfection; hybrid immunity (infection + vaccination) gives stronger, longer-lasting protection against severe disease.	10.1016/S1473-3099(22)00801-5
Berber E., 2024 (122)	Factors predicting COVID-19 vaccine effectiveness and longevity of humoral immune responses	Narrative review	Highlights the impacts of pre-existing immunity, comorbidities, and demographics like age and sex on influencing vaccine effectiveness.	10.3390/vaccines12111284
Petráš M., 2022 (123)	Risk factors affecting COVID-19 vaccine effectiveness identified from 290 cross-country observational studies until February 2022	Meta-analysis and meta-regression	Identified age, variant, time since vaccination, and high-risk status as key factors affecting vaccine effectiveness.	10.1186/s12916-022-02663-z
Ioannidis J. P. A., 2022 (124)	Factors influencing estimated effectiveness of COVID-19 vaccines in non-randomised studies	Narrative review	Discusses multiple factors that may bias and/or confound estimates of vaccine effectiveness and proposes ways in which certain limitations can be overcome.	10.1136/bmjebm-2021-111901
Ssentongo P., 2022 (125)	SARS-CoV-2 vaccine effectiveness against infection, symptomatic and severe COVID-19	Systematic review and meta-analysis	Vaccine effectiveness declined in older adults above 65 years of age; effectiveness also varied by vaccine platform.	10.1186/s12879-022-07418-y

Due to insufficient data, the true effectiveness of COVID-19 vaccines in the African population cannot be ascertained. According to the International Vaccine Access Centre (IVAC), only 8 out of 686 studies (1.2%) published or reported as pre-prints on COVID-19 vaccine effectiveness have been conducted in Africa (15). To address this disparity, the World Health Organization (WHO) launched an African Region Monitoring Vaccine Effectiveness (AFRO-MoVE) Network, which notably supported COVID-19 vaccine effectiveness studies in several African countries (16); although at the time of writing, findings from these studies had not yet been published. Nonetheless, it is also important to note that, due to distinct vaccine diplomacy efforts across all African countries, each country received and administered different vaccines which could limit generalizability of findings from vaccine effectiveness studies across the continent. Some countries also administered multiple different vaccines which complicates interpretability and analysis within countries too. Regardless, this data on the effectiveness of different vaccines in African countries can offer important insights to inform public health policies and can inform future vaccine research and development.

**Figure 2:**
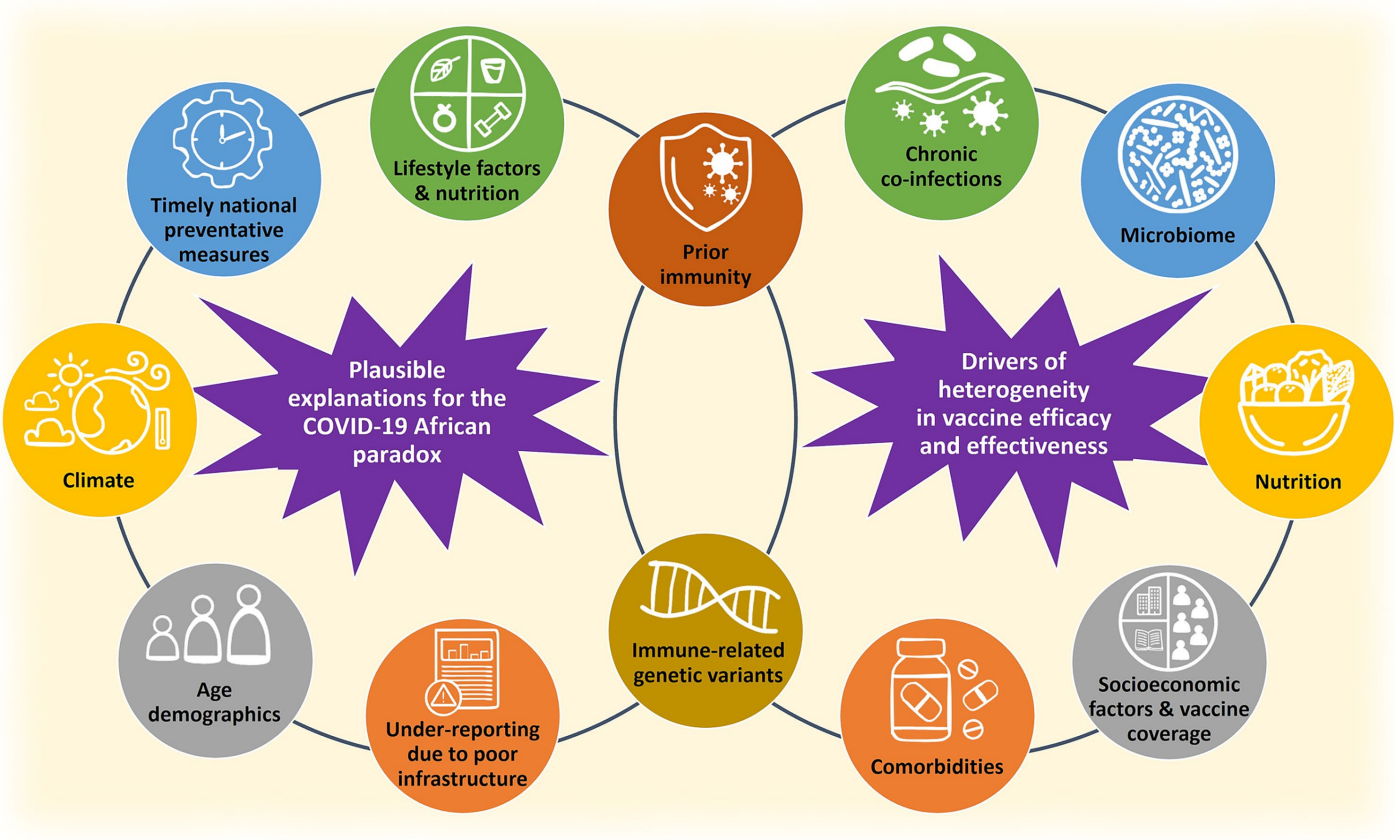
A summary of potential factors which could explain the reduced burden of COVID-19 and heterogenous vaccine responses in Africa.

As summarized in Figure 2, this review firstly evaluates hypotheses proposed and studied in the published literature to explain the COVID-19 African paradox. Secondly, it aims to explore factors that may influence heterogenous responses to vaccines in the African population. Knowledge gaps which limit our understanding of this paradox and heterogeneity in vaccine efficacy will be highlighted and discussed in the broader context of managing outbreaks caused by novel pathogens and enhancing pandemic preparedness, particularly for Africa. This is especially important to inform foundational research and preparatory efforts in vaccine development that could, when necessary, support the 100-day mission of developing an effective vaccine for a new pandemic threat in 100 days (17).

As this is a narrative (non-systematic) review, a predefined search strategy or formal inclusion and exclusion criteria were not employed. Relevant literature contributing insights into the conceptual understanding of highlighted topics and field of interest was identified through searches of PubMed, Google Scholar, and where appropriate, grey literature from reputable and credible sources.

## 2 Plausible explanations for less severe SARS-CoV-2 health outcomes in Africa

### 2.1 Underreporting as a consequence of poor healthcare infrastructure

A commonly accepted hypothesis relates to many cases and deaths being undetected and unreported in this population due to a lack of efficient healthcare systems and limited access to resources in healthcare facilities (18). However, many African countries, such as Uganda, Kenya, Rwanda, Cameroon, Zambia, South Africa, and Botswana, made early efforts supported by the Africa Centre for Disease Control (Africa CDC) to strengthen their capacities in the face of the pandemic. These efforts included training healthcare personnel, procuring testing kits to enhance screening and isolation programs, and investing in basic preventative solutions, such as hand sanitizers, which were made readily available to the public. Emphasis was also put on effectively communicating good hygiene and safety guidelines through various social media channels and displaying of posters in public spaces (19).

The hypothesis of case underreporting was initially tested via *in silico* models. An epidemiological model revealed notable differences in levels of case underreporting across various countries in Africa. Whilst the average rate of SARS-CoV-2 case reporting in Africa was estimated to be 5.37%, Libya had the highest rate of 30.41%, and São Tomé and Príncipe had the lowest rate of 0.02% (20). Population-based models estimated that actual infection rates in Africa were no less than the rest of the world, and could potentially be higher (21). These results were supported by a meta-analysis which pooled 153 SARS-CoV-2 seroprevalence studies done in African populations. It reported that despite reports of substantially low case numbers, population levels of SARS-CoV-2 specific antibodies had significantly increased during the first year of the pandemic (22). This implied that a higher proportion of the population had been infected than reported in Africa, and challenged the notion of the African population being less susceptible to SARS-CoV-2 infection. Additionally, another study estimating excess mortality due to COVID-19 claimed that death underreporting was especially greater in sub-Saharan Africa than in other regions (23). However, the WHO Africa Regional Office and their collaborators disagreed with these findings (24). Their observations and analyses indicated that the under-reporting rate in Africa was comparable to the rest of the world.

However, it is also important to consider that results from serological tests can vary by commercial assay kits used and may not be directly comparable (25). Additionally, *in silico* modelling studies have a major limitation in that their predictions can be significantly skewed by a low accuracy of training parameters and baseline figures used, and the relative contributions of confounding variables accounted for (26).

Nonetheless, a statistical analysis that controlled for underreporting showed that Africa still saw fewer COVID-19 cases and deaths by a factor of 2.7 and 1.4 respectively, compared to all other continents (27). Due to a lack of data on excess mortality in Africa during the pandemic resulting from weak death registration systems, it is difficult to conclusively interpret SARS-CoV-2 mortality in the continent to ascertain whether the disease burden was genuinely lower, particularly in the older population.

### 2.2 Timely implementation of preventative measures nationally

Africa was the last continent to see its first case of SARS-CoV-2 infection, which was reported from Egypt on the 14th of February 2020 (28). It hence had the advantage of learning from the experiences of regions affected earlier, and imposing mitigation strategies in advance, unlike many other parts of the world which were caught unprepared. Moreover, controlling infectious disease epidemics was not a novelty for the African continent, as it had in recent years experienced managing multiple outbreaks, including those caused by Ebola (29) and chikungunya viruses (30).

Many African countries seized the early opportunity and adapted previous outbreak management plans to contain SARS-CoV-2. They began early surveillance at borders, restricted travel, and upon detection of the first few cases, imposed mandatory quarantines, national lockdowns, and curfews to limit social gatherings (28). Africa CDC played a key role in monitoring and coordinating responses across the continent. It also supported capacity building and response planning to enhance preparedness at the face of the pandemic (19). There were however a few countries, such as Tanzania, which did not implement recommended protective measures to protect its community throughout the pandemic, as political leaders questioned scientific guidance and were hesitant to believe in the existence of SARS-CoV-2 (31).

Regardless, as the pandemic progressed, many African countries found it difficult to sustain stringent restrictions for prolonged periods of time due to various political, economic, and social reasons. By the second wave in late 2020, restrictions had been relaxed in most regions (32). Thus, mitigation strategies were likely effective in the early phase of the pandemic, but had limited contributions in maintaining an overall reduced burden in the long run, particularly in the face of fragmented health systems (27).

Furthermore, a modelling study has reported that the stringency or timing of government-imposed restrictions was not predictive of COVID-19 mortality during the first wave of the pandemic across different countries in Africa (33).

### 2.3 Climate

Africa may have benefited from its warmer climate which could have limited transmission of SARS-CoV-2. Higher temperatures have been noted to restrict transmission of other respiratory viruses, like influenza, whose cases have been observed for years to peak during winters (34).

Initial studies carried out towards the beginning of the pandemic found inverse correlations between temperature and number of COVID-19 cases. This suggested that colder climates favoured SARS-CoV-2 transmission (35, 36). These findings were supported by lab studies showing greater persistence and increased stability of human coronaviruses in lower temperatures and more humid conditions (37).

Contrastingly, more recent studies have found that temperature and climate in isolation are not sufficient to significantly reduce SARS-CoV-2 transmission. It is a more complex interplay of weather, human behaviour and mobility that influences transmission rates. Individuals tend to stay indoors to keep warm in winter and minimize natural ventilation, this significantly enhances and favours virus transmission cycles (38, 39). Nonetheless widespread transmission was observed even in tropical regions with warmer climate.

### 2.4 Lifestyle factors and nutrition

Despite widespread transmission of SARS-CoV-2 across all regions, epidemiological studies have reported more cases being observed from urban areas compared to rural regions (33, 40). A study conducted in Kenya has also shown that living in urban areas compared to rural regions was associated with higher levels of pro-inflammatory cytokines and chemokines (41). Although access to healthcare resources and facilities may be limited in rural areas, it may be easier to disrupt transmission cycles in these regions which tend to have a lower population density (40). Moreover, a key aspect of the lifestyle of rural populations is that individuals spend the majority of their time outdoors (42), which further reduces chances of viral spreading (43). As Africa is the least urbanized continent with more than half of its population living in rural counties (44), it may have benefited from this geo-epidemiological profile.

Additionally, due to the more physically demanding nature of jobs in rural areas, and the consumption of traditional African heritage diets comprising of locally produced fresh foods, rural African populations may be at a lower risk of developing non-communicable diseases such as hypertension, diabetes, obesity, respiratory diseases, and cardiovascular diseases compared to Westernised populations, which have a more sedentary lifestyle and consume mostly ultra-processed foods (45, 46). However, food insecurity is still a significant issue in many African regions (47) with a high prevalence of malnutrition that increases vulnerability to infections (48). Nonetheless, these are important considerations to examine when assessing susceptibility of the African population to severe COVID-19, since non-communicable diseases have been described to be strong predictors of severe SARS-CoV-2 infection outcomes (49, 50).

### 2.5 Immune-related genetic variants

The variation in immune-related host genetics within and between African populations may have also contributed to the heterogeneity in susceptibility to COVID-19, as has previously been noted with multiple infectious diseases including malaria, HIV, and schistosomiasis (51, 52). Severe COVID-19 has been associated with multiple genetic variants through genome-wide association studies (GWAS) (53). One such variant relates to the ABO blood group O having the lowest risk (54). In a study conducted in Nairobi on blood donors, this blood group was found to be more prevalent amongst indigenous African donors (49%) compared to the general donor population (45%) and Asian donors (34%) (55).

Importantly, SARS-CoV-2 internalises into host cells by binding to angiotensin-converting enzyme 2 (ACE2) receptors, but ACE inhibitors have been shown to be less effective in treating high blood pressure in patients of African descent (56) due to reduced expression of the ACE2 protein (57). This may subsequently have naturally reduced the likelihood of these individuals becoming infected, as well as limited progression of infection. Variations in human leukocyte antigens (HLA) have also been shown to influence the nature of immune responses, with certain types of HLA inducing activation of inflammatory immune pathways, while others reduce inflammatory responses (58). To the best of our knowledge, HLA types have not been mapped at the population level in Africa and so the impacts of prevalent HLA types in the context of SARS-CoV-2 infection in the African population are unclear.

Contrastingly, some studies have found that individuals of African descent residing in Europe or the United States were at an increased risk of progressing to severe stages of SARS-CoV-2 infection (59, 60). These findings imply that genetic factors are unlikely to have contributed significantly to reducing susceptibility of the African population to severe COVID-19, as if this were the case, then African descendants residing outside the continent would similarly have benefited from a reduced susceptibility. However, in England and Wales, this higher risk for African descendants has been correlated with increased exposure due to other factors that can enhance transmission of SARS-CoV-2, such as occupation and overcrowding in larger households (61). Therefore, whether immune-related host genetics played a key role in influencing outcomes of SARS-CoV-2 infection is still ambiguous.

### 2.6 Age demographics

In the early days of the pandemic, advanced age was quickly identified as a significant risk factor for severe COVID-19 (62). In contrast to the ageing population in many westernised countries, the majority of Africa’s population is youth. Hence, the population overall may have been less susceptible to severe disease, with a high proportion of infections being asymptomatic or only causing mild disease (40, 63).

Modelling studies, which aimed to understand the role of Africa’s demographics in shaping outcomes of SARS-CoV-2 infection, have strongly supported the hypothesis that low reports of severe disease and deaths from Africa are a result of its younger population (27). That is despite high infection rates, implying that the primary advantage was that of attenuated disease severity not reduced susceptibility (40). The observed outcomes of SARS-CoV-2 infection were even lower than that predicted by age-based models (6). This is because the calculations were derived from infection fatality rates determined from higher income countries. Since the health systems in these countries were overwhelmed, the calculations would have predicted a higher number of severe cases than was observed. This subsequently implies that age alone cannot explain the reduced burden of disease in Africa.

### 2.7 Prior immunity

Data curated by the WHO in 2021 revealed that infectious diseases, particularly lower respiratory infectious diseases, malaria, and tuberculosis, were amongst the top five leading causes of death in Africa (64). This highlights the constant exposure to endemic pathogens in the African continent. Hence, through regularly battling with endemic infections, which can result in the acquisition of cross-reactive protection and broadly neutralising antibodies, the population’s immunity may have been better trained to recognise and rapidly clear new infections such as those caused by SARS-CoV-2.

Seroprevalence studies found cross-reactive antibodies to SARS-CoV-2 from previous exposures to human coronaviruses in both African and European cohorts (65, 66). However, the diversity of heterologous immunity may be wider in the African population due to their greater exposure to endemic pathogens (67). For example, patients infected with SARS-CoV-2 had reactivation of *Plasmodium falciparum* specific B- and T- cells depending on the severity of disease (68). This may be attributed to pre-existing cross-reactive protection (67), but may also be a result of pre-trained regulation of regulatory, pro-, and anti-inflammatory immune responses from previous malaria infections (69). This is because malaria also overstimulates the immune system (70). Thus, individuals from endemic regions may have a higher tolerance to pro-inflammatory immune responses, and may be less susceptible to succumb to severe immunopathology induced by progressive SARS-CoV-2 infection. Further research is needed to elucidate this theory.

It is also essential to take into account the fact that serology studies primarily tested for antibody titers to SARS-CoV-2 spike proteins. Although the highest homology of the SARS-CoV-2 spike protein is to the spike proteins from SARS-CoV-1 (76%) and MERS-CoV (35%) (71), these viruses have not been found to circulate in Africa (72). Hence any pre-existing cross-reactive antibodies in this population are likely from their constant exposure to other known and unknown endemic pathogens (67).

Furthermore, live vaccines such as the Bacillus Calmette-Guerin (BCG) vaccine are mandated in many African countries to protect against tuberculosis. Such routine vaccinations could also lead to the development of heterologous immunity by inducing long-lived non-specific responses that can offer broad protection against more infectious agents including SARS-CoV-2 (73). Hygiene and sanitation practices in low- and middle-income countries also tend to be less rigorous than in westernised regions, which further exposes individuals to more microbes and expands commensal microbial communities throughout their lifetime. As implied by the “hygiene hypothesis,” this contributes to enhancing the diversity of immune repertoires and priming immune responses to detect new antigens (74).

## 3 Potential drivers of heterogeneity in vaccine efficacy and effectiveness in Africa

### 3.1 Chronic immunosuppressing co-infections

As mentioned earlier, the African continent carries the highest burden of infectious diseases globally. Sub-Saharan Africa has the highest global prevalence of the immune-modulating pathogen HIV (75) and parasitic helminths like *Schistosoma mansoni* (76). Ongoing chronic infections with these pathogens suppress the immune system and may influence both the efficacy and effectiveness of vaccines in all populations in endemic regions. Whilst HIV directly infects and depletes CD4 + T-cells (77), *S. mansoni* indirectly modulates the immune system by expressing and secreting immunoregulatory proteins which suppress Th1 immune pathways and promote Th2 responses (78). Vaccines, particularly those directed towards eliciting cellular immune responses, are therefore unlikely to induce the desired protective immune response in those infected with these parasites. A number of studies have in fact reported attenuated responses to various vaccines in individuals infected with HIV (79) and in those repeatedly exposed to helminth infections (80).

### 3.2 Nutrition

Africa bears the greatest burden of malnutrition, particularly in children under 5. Cases of malnutrition continue to rise and present significant public health challenges for these nations (48), including impaired immune responses to vaccines. Specifically, deficiencies in micronutrients such as calcium, potassium, folic acid, iron, zinc, selenium, and vitamins A, E, K, and D have previously been associated with attenuated pneumococcal vaccine-induced immunity (81). More recently, studies have also found that COVID-19 vaccinations induce weaker antibody responses in those who are underweight and malnourished (82).

As the prevalence of malnutrition is relatively higher in Africa compared to all other continents, and the efficacy of multiple vaccines, such as those against pneumococcus, rotavirus, polio, cholera amongst others, have been found to be low in malnourished individuals (83), the overall effectiveness of vaccines may be reduced in Africa.

### 3.3 Comorbidities

Anaemia, a common comorbidity resulting from malnutrition and iron deficiency, is of particular importance for the African population. It has been shown to limit immune responses to many vaccines including those against pneumococcus, diphtheria, pertussis, and measles (84). In Africa, the prevalence of anaemia is greatest in children under 5 years of age, hence vaccine effectiveness may be greatly reduced in this vulnerable population.

With increasing urbanisation leading to more sedentary lifestyles and greater consumption of ultra-processed foods, the burden of non-communicable diseases continues to pose a major global health challenge, especially in many developing countries in Africa (85). Examples of such comorbidities include chronic liver disease, diabetes, hypertension, and coronary heart disease. Even though treatments for many of these conditions are available and can limit complications, healthcare is suboptimal in Africa and many face barriers of access due to healthcare costs (86). Because these diseases are known to weaken the immune system, they may also affect the level of protection induced by vaccines, especially when disease progression is not halted by effective treatment. Advancements in vaccine technology have however enhanced vaccine-induced immunity for patients living with these comorbidities (87). mRNA and viral vector COVID-19 vaccines validated this argument as they were found to be equally safe and effective in those with or without comorbidities (88).

### 3.4 Microbiome

The human microbiome, particularly the gut microbiome, has increasingly been shown to play a role in modulating the immune system (89). This implies that differences in microbiome composition between individuals and populations could drive varied responses to vaccines. Although the vast majority of studies characterising the human microbiome primarily exclude individuals from the Global South (90), there is some recent evidence suggesting that the microbiome of the African population is more diverse, and is composed of different proportions of varied taxa (91). The diversity and composition of the microbiome also varies across regions within Africa (92).

Given the gut microbiome’s direct role in the absorption and processing of oral vaccines, most experimental studies on mouse models and human population-based intervention studies have examined the gut microbiome’s impact on the efficacy and effectiveness of vaccines administered orally. These studies have shown significant differences in responses to oral vaccines across different geographical locations such as Ghana, Pakistan, and Bangladesh that can be attributed to diverse gut flora (93).

Responses to vaccines administered through alternative injectable routes have also been shown to be influenced by relative compositions of the gut microbiome (94–96). However, these studies only show associations, further studies are needed to determine causality and elucidate potential mechanisms by which the host microbiome can impact immune responses to vaccines.

### 3.5 Socioeconomic factors and vaccine coverage

Effectiveness of vaccines has been observed to be poorer in low- and middle-income countries and in rural regions (87, 97). Vaccine effectiveness calculations in these regions may however be skewed by limited coverage (98) which may be attributed to multiple factors, including affordability and access to vaccines (87). Since governments have limited budgets to acquire vaccines for their states, vaccination programmes may primarily be targeted towards high-risk, vulnerable populations who are more likely to experience severe disease. This would inadvertently reduce vaccine effectiveness estimates. Healthcare infrastructure in these areas is also often fragmented which may negatively impact vaccination programmes by further reducing coverage (99). Lack of appropriate storage facilities in remote rural regions further compromises vaccine potency and efficacy.

Literacy levels, which tend to be globally the lowest in sub-Saharan Africa (Our World in Data), also influence vaccine uptake. Individuals educated to a higher level and on the importance of vaccine uptake are more likely to accept and be willing to participate in vaccination programmes (100, 101).

### 3.6 Immune-related genetic variants

Variation in immune-related genes within and between populations can influence efficacy of vaccines across different populations. For example, heterogeneity in HLA types and toll-like receptors (TLRs) have been found to induce varied responses to different types of vaccines (102–104). This is because HLA molecules play a key role in directing immune responses by presenting epitopes to T-cells and inducing T-cell activation, whilst TLRs recognise foreign antigens and stimulate initial responses by the innate immune system. As different HLA types and TLR polymorphisms differentially recognise the same epitopes, vaccine constructs may be more or less immunogenic across different populations.

A study found additional polymorphisms in other genes beyond those encoding for HLA types which influence antibody responses to COVID-19 vaccines. These include TP53, ABO, APOE, ACE2, and CRP-related genes (105).

Studies investigating dynamics of vaccine-induced immunity across different ethnic groups and ancestry have also found that both cellular and humoral immune responses subsequent to measles and COVID-19 vaccines vary significantly by ethnicity (106, 107). This heterogeneity has been supported by a study showing significant differences in pre-vaccination gene expression profiles of myeloid and B-cell specific genes between African Americans and European descendants (108).

### 3.7 Prior immunity

Pre-existing immunity acts as a double-edged sword, as it can both positively and negatively influence the efficacy and effectiveness of vaccines. Factors such as timings between infection and vaccination, and antigenic divergence can influence whether prior immunity is beneficial or detrimental. Table 2A summarises studies on various vaccines against different pathogens which have shown pre-existing immunity to either protect against reinfection, or to augment vaccine effectiveness; whilst Table 2B summarises key studies which have observed and reported negative outcomes of pre-existing immunity on vaccine efficacy and effectiveness.

**Table 2:** Studies reporting effects of pre-existing immunity on vaccine effectiveness.

Study (first author, year)	Study title	Study type	Summary of study findings	DOI (for source verification)
(A) Positive effects				
Velázquez F. R., 1996 (126)	Rotavirus infection in infants as protection against subsequent infections	Cohort study	Natural rotavirus infection provides progressive protection against subsequent infection and severe disease.	10.1056/NEJM199610033351404
Rogawski. E. T., 2018 (127)	Quantifying the impact of natural immunity on rotavirus vaccine efficacy estimates	Clinical trial and simulation study	Previous natural rotavirus infection in children provides greater protection and can enhance vaccine efficacy.	10.1093/infdis/jix668
Levine M. M., 1981 (128); Pasetti M. F., 2012 (129)	Duration of infection-derived immunity to cholera; Insights from natural infection-derived immunity to cholera instruct vaccine efforts	Human challenge study; commentary article	Infection-derived immunity to cholera is long-lasting; this formed the basis of designing oral live vaccines to induce natural immunity.	10.1093/infdis/143.6.818;10.1128/CVI.00543-12
Kim J. H., 2016 (130)	Prior infection with influenza virus but not vaccination leaves a long-term immunological imprint that intensifies the protective efficacy of antigenically drifted vaccine strains	Experimental immunology study on a mouse model	Prior influenza infection, more than prior vaccination in some contexts, can improve the magnitude and breadth of responses to subsequent variant vaccines.	10.1016/j.vaccine.2015.11.077
Spinardi J. R., 2023 (131)	Hybrid immunity to SARS-CoV-2 from infection and vaccination—evidence synthesis and implications for new COVID-19 vaccines	Narrative review	Hybrid immunity derived from a combination of previous infection and vaccination confers stronger and broader protection against reinfection and severe disease than vaccination or infection alone.	10.3390/biomedicines11020370
Andrade A. G., 2023 (132)	Natural and hybrid immunity: a comparative study of T cell response against SARS-CoV-2	Case–control comparative immunological study	Vaccines complement pre-existing immunity from natural infection by orchestrating a more proficient antiviral immune response with an immuno-regulatory capacity.	10.1016/j.clicom.2025.07.001
Bobrovitz N., 2023 (121)	Protective effectiveness of previous SARS-CoV-2 infection and hybrid immunity against the omicron variant and severe disease	Systematic review and meta-regression	Individuals with hybrid immunity from both natural infection and vaccination had the highest magnitude and durability of protection.	10.1016/S1473-3099(22)00801-5
(B) Negative effects				
Ali M., 2011 (112)	Natural cholera infection–derived immunity in an endemic setting	Cohort analysis	Efficacy of vaccines against cholera is lower in endemic settings due to the presence of substantial long-lasting protection from natural infection.	10.1093/infdis/jir416
Bradt V., 2019 (133)	Pre-existing yellow fever immunity impairs and modulates the antibody response to tick-borne encephalitis vaccination	Clinical case–control study	Pre-existing immunity from yellow fever vaccinations impaired neutralizing antibody activity against tick-borne encephalitis virus—described as original antigenic sin as both are flaviviruses.	10.1038/s41541-019-0133-5
Santos-Peral A., 2024 (134)	Prior flavivirus immunity skews the yellow fever vaccine response to cross-reactive antibodies with potential to enhance dengue virus infection	Clinical cohort	Prior flavivirus immunity derived from vaccination against tick born encephalitis virus skews immune responses against subsequent yellow fever vaccination towards more cross-reactive antibodies with a lower neutralizing potential and can enhance dengue virus infection.	10.1038/s41467-024-45806-x
Zarnitsyna V. I., 2016 (135)	Multi-epitope models explain how pre-existing antibodies affect the generation of broadly protective responses to influenza	Mathematical modelling	Pre-existing antibodies from previous infection and vaccinations mask antigens which prevents effective boosting of protective responses.	10.1371/journal.ppat.1005692
Monto A. S., 2017 (136)	The doctrine of original antigenic sin: separating good from evil	Narrative review	Highlights studies showing that secondary exposures to different strains of influenza and to influenza vaccinations result in an attenuated immune response.	10.1093/infdis/jix173
Roy S., 2020 (137)	Impact of pre-existing immunity to influenza on live-attenuated influenza vaccine (LAIV) immunogenicity	Narrative review on pre-clinical and clinical studies	Pre-existing antibodies limit replication of live attenuated influenza vaccines and subsequent induction of T cells; hence reducing vaccine effectiveness.	10.3390/vaccines8040683
Xie Y., 2023 (138)	Immune interference in effectiveness of influenza and COVID-19 vaccination	Narrative review	Reviews evidence on the role of prior immunity from repeated exposure to influenza and COVID-19 either via natural infection or multiple vaccinations on the effectiveness of subsequent vaccinations.	10.3389/fimmu.2023.1167214
Wrynla X. H., 2025 (110)	Immune imprinting and vaccine interval determine antibody responses to monovalent XBB.1.5 COVID-19 vaccination	Case–control clinical immunogenicity	Pre-existing immunity affects humoral immunity elicited by an updated variant vaccine as antibodies against the original virus were boosted over variant-specific antibodies from the updated vaccine.	10.1038/s43856-025-00898-4
Fausther-Bovendo H., 2014 (139)	Pre-existing immunity against Ad vectors: humoral, cellular, and innate response, what’s important?	Narrative review	Prior immunity against vaccine vectors can neutralize and clear the vector which subsequently reduces overall vaccine immunogenicity and reduces vaccine efficacy.	10.4161/hv.29594

Pre-existing immunity can enhance vaccine effectiveness, as vaccination can further boost pre-existing protection. This benefit is pronounced with longer time intervals between exposures (109, 110). However, due to a higher level of baseline protection present in populations with pre-existing heterologous immunity, the relative gain from vaccination is lower compared to naïve populations. This thus results in relatively lower vaccine effectiveness in populations with prior protective immunity (111). This is accentuated in scenarios where pre-existing immunity provides even greater protection than vaccinations. For example, natural cholera infection has been found to induce long-lasting protection that significantly reduces the severity of disease in subsequent infections, and is more protective than vaccination (112).

However, pre-existing cross-reactive protection can also compromise the efficacy of vaccines. Immune interference, which can be caused by immune imprinting from previous infections and vaccinations, may limit the extent to which one’s immune system can be primed by new vaccinations to protect against emerging pathogens (14). Immune imprinting refers to a phenomenon whereby immune memory from initial exposures to similar immunogens is preferentially reactivated, even though it may not be the most appropriate for subsequent interactions with similar but unrelated immunogens. Hence, vaccines with antigens that are very similar to those that individuals have previously been exposed to, are likely to fall victim to immune interference (113, 114). As highlighted in Table 2B, this phenomenon has previously been observed and reported for vaccines against different pathogens, including recent COVID-19 vaccines.

## 4 Discussion

In spite of the WHO officially declaring the end of the COVID-19 pandemic on the 5th of May 2023, there are still many lessons to learn from it. For example, understanding the disproportionate burden throughout the world can provide invaluable insights on immune responses to novel pathogens and future pandemic preparedness. It can also inform scientific research, the design of population-based mitigation strategies, and development of targeted public health policies in preparation for future outbreaks.

As pointed out through the first half of this review, multiple hypotheses have been proposed to explain the skewed outcomes observed in Africa throughout the COVID-19 pandemic, but many justifications remain inconclusive due to knowledge gaps. A considerable amount of variability in disease outcomes has been observed from countries within the continent too (6, 7), yet limited studies account for this. Through investigating the role of various demographic and socioeconomic factors in influencing the variability in detection of first COVID-19 cases and mortality across different countries in Africa, urbanisation, international connectivity, and HIV prevalence were identified as strong predictors of worse COVID-19 outcomes (33). As it is more likely that a combination of multiple factors led to the attenuated COVID-19 disease outcomes observed in Africa, such estimates of relative impacts of various factors provide invaluable contributions towards aligning points of prioritisation when planning future outbreak response frameworks.

Additionally, clinical immunology studies offer great insights into population vulnerabilities that not only inform response strategies during a pandemic but can help to develop frameworks for future outbreaks. However, comparison of multiple studies requires careful consideration to minimise biases that could arise due to many confounding variables. Such limitations include the use of different commercial antibody test kits and different vaccine types across different studies. This can be overcome by recruiting representative sample groups within studies and/or conducting multi-centre collaborative studies which assess responses in participants from various backgrounds in a controlled, standardised manner. Antibody responses have also primarily been assessed against the spike protein. Analysing immune responses to a wider range of antigens from the SARS-CoV-2 proteome may reveal important differences such as natural infection vs. vaccination signatures, in other words, immune response biomarkers of the vaccine. With these distinctions, not only can a more realistic burden of SARS-CoV-2 be estimated, but responses predictive of progression to severe disease may be identified, and effectiveness of different types of vaccines may be better elucidated.

An important immunological indicator of vulnerability to novel infections is the breadth and diversity of pre-existing heterologous immunity. Hence, through characterising baseline humoral and cellular immune profiles, and their influences on priming immune phenotypes, novel insights on population vulnerability to emerging pathogens can be revealed. It is especially important to characterise T-cell responses to understand differences in T-cell activation subsequent to both natural infection and vaccination. These cells have been shown to play a critical role in mediating immune responses to natural SARS-CoV-2 infection, but remain poorly characterised in African populations (115–117). Data on the prevalence of different HLA types across populations would also be crucial in informing population vulnerability, vaccine efficacy, and design of vaccine constructs.

Like attenuated COVID-19 outcomes, heterogenous responses to vaccines are likely to be a result of a combination of multiple factors, including the use of different types of vaccines in different regions. Understanding the role of each of the factors individually, and in combination, in influencing the efficacy and effectiveness of vaccines is important to not only inform vaccine research and development, but also to improve infrastructures around vaccination programmes and guide deployment strategies. As vaccines are the primary intervention sought to control infectious disease outbreaks, immunization policies developed rely strongly on vaccines being highly effective.

As described through the second part of this review, a number of factors have been associated with influencing vaccination outcomes, however, there is limited knowledge on how factors such as nutrition and the microbiome influence responses to vaccines. Nevertheless, it has recently been proposed that co-administration of mineral and vitamin supplements and pro-biotics may enhance vaccine efficacy (118–120). Such complementary solutions should thus be considered and investigated in the African population, and subsequently recommended if found to be effective.

To improve vaccine coverage, low- and middle- income countries need to be supported not only financially to secure more vaccine doses, but through capacity building programmes too, that can encourage local vaccine development and manufacturing. In the face of shrinking overseas budget aid, capacity building and empowering local talent is even more crucial to enable African countries to take ownership of developing and strengthening their healthcare systems. Setting up regional vaccine manufacturing hubs with local collaboration can significantly reduce costs related to vaccine acquisition for the continent (99). Scientific leaders in African countries should also be encouraged to adopt multidisciplinary and transdisciplinary approaches, and to engage and educate the public to enhance awareness of vaccines and their importance.

The role of pre-existing immunity on vaccine efficacy and effectiveness also needs to be better characterised during clinical trials and in real-world situations as part of pharmaco-vigilance programs, respectively. Moreover, immunological studies are needed to elucidate mechanisms and factors which direct either a positive or negative effect of prior immunity on vaccine efficacy. Knowledge from these studies is critical for informing vaccine research and development, and the design of vaccine effectiveness monitoring studies.

Overall, knowledge gaps discussed here highlight the need for more immunology and epidemiology studies to be conducted in the African population. During the early phases of an outbreak, whilst interventions are still being developed and deployed, these studies provide crucial insights that can inform development of context-appropriate mitigation measures to contain the spread of outbreaks. Findings from these studies can also help improve vaccine development and intervention deployment strategies by identifying vulnerable populations to prioritise. The importance of and need for strengthening healthcare systems in Africa cannot be over emphasised to overcome inequities relating to conducting research and accessing vaccines. As was observed through the COVID-19 pandemic, Africa faces significant delays in gaining access to these interventions. Even though the 100-day mission rightly aims to accelerate the development of diagnostics, therapeutics, and vaccines during the next pandemic (17), it is not sufficient on its own. These interventions also need to be globally effective and rolled out appropriately.

To conclude, SARS-CoV-2 serves as an instructive case study that provides lessons across the fields of immunology, epidemiology, and public health as it can enhance understanding on host immune responses to both novel pathogens and vaccines. The experience with SARS-CoV-2 helps to inform the development of globally effective vaccine platforms and pandemic preparedness frameworks. However, it is important to consider that a future pandemic may be caused by a different pathogen altogether. Hence, to better prepare for the unexpected, frameworks should be adaptable and scalable, building capacity for responses that can be adapted to a range of pathogens and environments.
